# Concentrations of oligosaccharides in human milk and child growth

**DOI:** 10.1186/s12887-021-02953-0

**Published:** 2021-10-30

**Authors:** Philipp Menzel, Mandy Vogel, Sean Austin, Norbert Sprenger, Nico Grafe, Cornelia Hilbert, Anne Jurkutat, Wieland Kiess, Aristea Binia

**Affiliations:** 1 Leipzig University, Leipzig University Hospital for Children and Adolescents, LIFE Child, Ph.-Rosenthal-Str. 27, 04103 Leipzig, Germany; 2 Center for Pediatric Research, Leipzig University, Department of Women and Child Health, Leipzig University, Liebigstr. 20a, 04103 Leipzig, Germany; 3 Nestlé Research, Société des Produits Nestlé SA, Route du Jorat 57, 1000 Lausanne, Switzerland

**Keywords:** Children, Growth, Human Milk oligosaccharides, HMO, Secretor status, FUT2

## Abstract

**Background:**

The relationship between human milk oligosaccharides (HMO) and child growth has been investigated only insufficiently with ambiguous results. Therefore, this study examines potential influencing factors of HMO concentrations and how HMO are associated with child growth parameters.

**Methods:**

Milk samples from the German LIFE Child cohort of healthy children were analyzed for 9 HMO. Putative associations with maternal and child cofactors and child height, head circumference and BMI between 3 months and 7 years of age were examined. Secretor status, defined as the presence of 2′-fucosyllactose, was investigated for associations with infant outcomes.

**Results:**

Our population consisted of 21 (14.7%) non-secretor and 122 (85.3%) secretor mothers. Maternal age was significantly associated with higher 3′SL concentrations; gestational age was associated with LNT, 6′SL and LNFP-I. Pre-pregnancy BMI was negatively associated with LNnT only in non-secretors. The growth velocity of non-secretors’ children was inversely associated with LNnT at 3 months to 1 year (*R* = 0.95 [0.90, 0.99], *p* = 0.014), 1 to 2 years (*R* = 0.80 [0.72, 0.88], *p* < 0.001) and 5 to 6 years (*R* = 0.71 [0.57, 0.87], *p* = 0.002). 2’FL was negatively associated with BMI consistently, reaching statistical significance at 3 months and 4 and 5 years. Children of non-secretors showed higher BMI at 3 months, 6 months, and 3, 6, and 7 years of age.

**Conclusion:**

We found that some associations between HMO and infant growth may extend beyond the infancy and breastfeeding periods. They highlight the importance of both maternal and infant parameters in the understanding of the underlying associations.

**Trial registration:**

The study is registered with ClinicalTrial.gov: NCT02550236.

**Supplementary Information:**

The online version contains supplementary material available at 10.1186/s12887-021-02953-0.

## Introduction

Human milk is being explored intensively to understand its composition and physiological role for the breastfed infant. Lipids [1, 2] have been identified as the most significant source of energy in mature milk. Additional important compounds of human milk are proteins, including enzymes and bioactive proteins like antibodies, nitrogenous compounds, and especially nucleotides, which influence the enzyme activity and the functionality of the immune system, hormones, vitamins, water and carbohydrates, including lactose and oligosaccharides. Together with lipids, lactose is an important source of energy, especially for the developing human brain [3]. Human milk oligosaccharides (HMO) form the third largest solid fraction in human milk; they represent about 20% of the total carbohydrates, with an estimated amount of up to 20 g/L in colostrum [4, 5]. HMO are composed of 5 different monosaccharides (Glc, Glucose; GlcNAc, N-Acetylglucosamine; Gal, Galactose; Fuc, Fucose; Neu5Ac, N-Acetylneuraminic acid), which are linked together via glycosidic bonds [6] to produce a wide variety of different structures [7].

HMO have been investigated for their potential role in the early growth of neonates; however, their effects in early and later metabolic health are unclear [8–15]. To date, only 1 study has investigated the association between HMO and growth beyond infancy [12]. The possible underlying biochemical or physiological processes linking HMO and infant growth are not understood. HMO are indigestible but can be fermented at least partly by the infant’s microbiome [16–18]. Thus, they support the maturation of the gastrointestinal tract and the immune system and can protect against the colonization of pathogenic microorganisms by inhibiting their anchoring to human epithelial cells [19–22]. In preclinical models, various studies have examined the effects of HMO (sometimes combined with microbiota) on gut epithelial maturation, differentiation and signaling processes [23–25], which can affect nutrient uptake and developmental programming, as exemplified by their effects on bone formation [26]. HMO concentrations are influenced by lactation stage and maternal genetic factors [27–30] and probably to a lesser extent by maternal weight and body mass index (BMI) before pregnancy [19, 27, 28].

Therefore, in this study, we aim to assess the association between the oligosaccharide composition in breast milk at 3 months postpartum and a) maternal factors (maternal age, pre-pregnancy BMI), b) the child’s birth parameters (anthropometric measurements, gestational age (GA)) and c) the subsequent growth until the age of 7 years (height, growth velocity, head circumference (HC), BMI). According to the literature, we hypothesize only weak associations between HMO concentrations and the maternal factors. Further, we expect associations between HMO and the child’s anthropometric measurements, with higher effects in the first year of life and lower effects for older ages.

## Methods

### Study design and participants

All data were collected within the LIFE Child study at the Research Centre for Civilization Diseases in Leipzig, Germany (www.ClinicalTrial.gov: NCT02550236). Children and their parents have been recruited from the 24th week of gestation to 16 years of age to investigate environmental, metabolic and genetic associations with children’s development [31]. The study is described in detail elsewhere [31, 32].

**Fig. 1:**
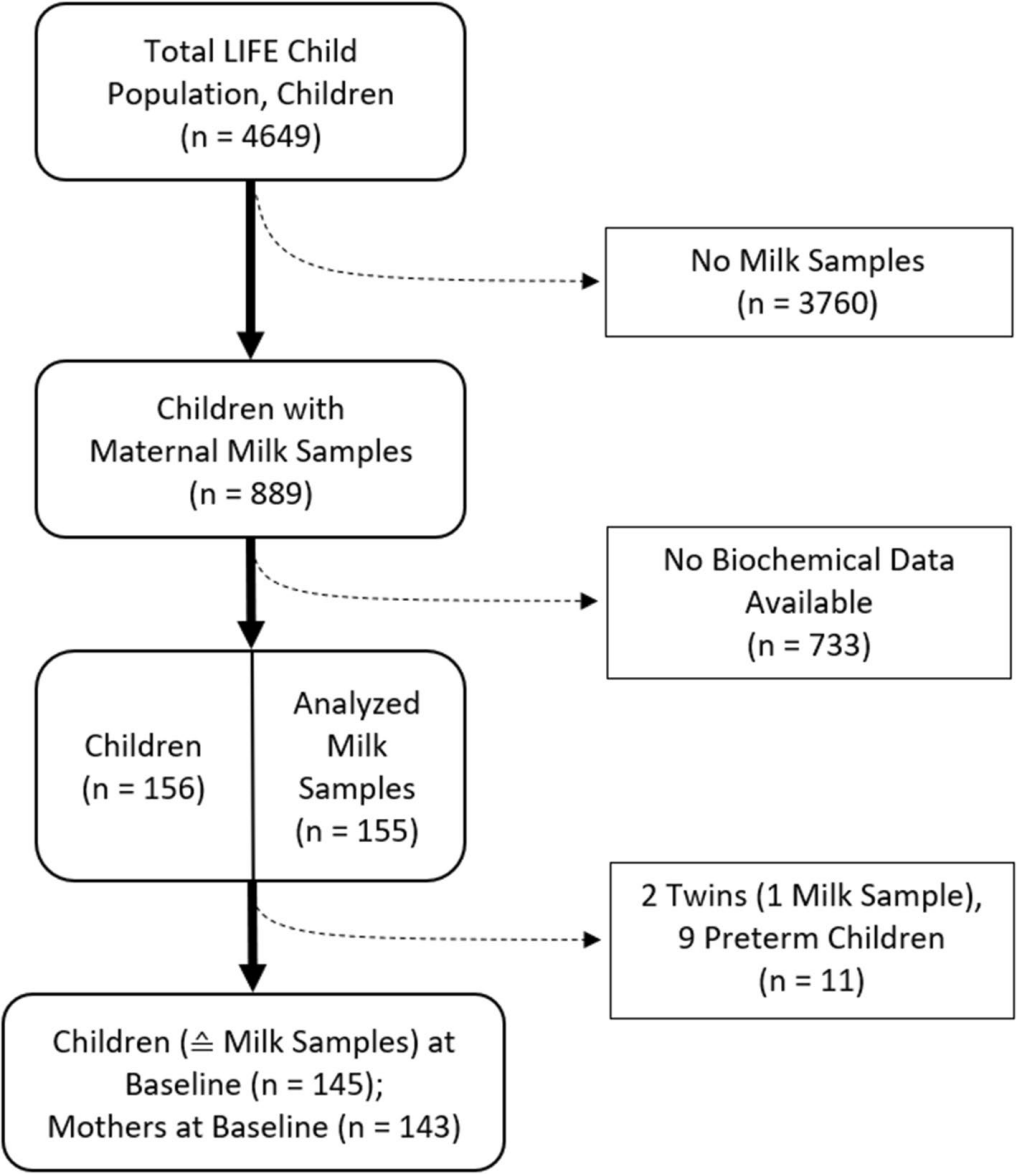
Flow chart of the study design

Between 2011 and 2014, 155 milk samples were collected from 153 mothers who nurse at the 3-month baseline visit (Fig. 1). The 3-month-visit took place after their 2nd full month and before the end of the 1st week of their 4th month of life. We excluded 1 sample because of a twin pregnancy and 9 samples because of preterm birth. Two mothers contributing 2 pregnancies were included. Finally, 145 sample-children combinations were included. Further, 132 (91%) follow-up measurements were documented at 6 months, 122 (84%) at 1 year, 106 (73%) at 2 years, 104 (72%) at 3 years, 90 (62%) at 4 years, 87 (60%) at 5 years, 59 (41%) at 6 years, and 37 (26%) at 7 years of age.

Informed written consent was provided by all parents for their children, participation in each procedure were voluntary. The study has been conducted per the Declaration of Helsinki. The study protocol was approved by the Ethics Committee of the Medical Faculty of the University of Leipzig (Reg. No. 264-10-19042010).

### Milk collection, storage and analysis

Under private conditions, about 20 mL of milk was collected at the 3-month visit using a milk pump (Medela Symphony®). The samples were obtained during the morning before lunchtime (1 pm). The first 20 mL was used. The milk was within at most 20 min stored without processing at − 80 °C at our biobank [31] until its transport to Nestlé Research, Lausanne, on dry ice. The concentrations of 9 HMO were determined by liquid chromatography with fluorescence detection following a previously described validated method [33]. The calibration standards of 2′-fucosyllactose (2’FL), 3-fucosyllactose (3-FL), 3′-sialyllactose (3′SL), 6′-sialyllactose (6′SL), lacto-N-tetraose (LNT), lacto-N-neotetraose (LNnT), lacto-N-fucopentaose-I (LNFP-I), lacto-N-fucopentaose-V (LNFP-V) and lacto-N-neofucopentaose (LNnFP) were purchased from Elicityl (Crolles, France), where the HMO content of the standard powders was determined by quantitative nuclear magnetic resonance spectrometry. For each HMO, 9 point calibration curves were prepared in the ranges 2’FL: 10–4900 mg/L, 3-FL: 10–2800 mg/L, 3′SL: 2–900 mg/L, 6′SL: 2–900 mg/L, LNT: 4–1900 mg/L, LNnT: 2–1000 mg/L, LNFP-I: 4–2000 mg/L, LNFP-V: 2–900 mg/L, LNnFP: 2–900 mg/L. With each batch of analysis (or every 25 samples if batches were larger) a reference pooled human milk sample (Lee Biosolutions, Maryland Heights, USA) was analyzed to ensure the method performance remained consistent between days and between batches of analyses.

### Measurements

Maternal pre-pregnancy weight and height and the child’s birth parameters were taken from the maternity log (“Mutterpass”) [34]. This booklet is updated by medical staff during pregnancy checkups.

Measurements were taken by trained research assistants according to standard procedures. Height/length was measured using the Dr. Keller II infantometer until 1 year of age and the Dr. Keller I stadiometer afterward. Weight was determined by using a scale (the seca 757 or the seca 701). HC was measured using a non-dilatable measuring tape. BMI was calculated. Height/length, weight, and BMI were transformed to standard deviation scores (SDS) according to the guidelines from the German Working Group on Obesity in Childhood and Adolescence [35]. HC measurements were transformed to SDS using German standards from the KiGGS study [36]. As a measure of growth, growth velocity was calculated as the standardized difference between the 3-month height and the 1-year height of the child and afterward between 2 consecutive height measures.

### Statistical analyses

All statistical analyses were carried out using R 4.0 [37]. Descriptive statistics were given as median [Q1;Q3] for HMO and mean (standard deviation) for the other continuous variables (Table 1). For all HMO, values below the limit of quantification (LoQ) were set to the LoQ and marked as left censored. Correlations (r) between specific HMO were investigated using Pearson correlations on the log-transformed values. Secretor status was determined based on the 2’FL concentration, with values below the LoQ of ≤53 mg/L corresponding to mothers who are non-secretors (NSM) and > 53 mg/L corresponding to mothers who are secretors (SM) [30, 33]. For HMO, differences in medians between SM and NSM were tested using Kruskal-Wallis tests or censored regression models when data below the LoQ occurred. For the other continuous variables, differences in means were tested using t-tests. Chi-squared tests were applied to test differences in proportions. The associations between the HMO and the children’s and mothers’ parameters were examined using generalized additive models for location, shape, and scale [38, 39], with HMO as the outcome and the other variables as predictor variables. Modeling was done separately for each age group, assuming a log-linear relationship between predictor and outcome. Due to the HMO values’ considerable skewness, these values were log-transformed. A Box-Cox Cole and Green distribution or its censored equivalent was chosen to describe the outcomes’ distributions. Investigating the models’ error structure revealed variances according to secretor status. Therefore, variance was modeled dependent on secretor status. Skewness was dependent on secretor status only for LNnT. With evidence of an interaction between the predictor and the secretor status, the respective interaction term was included in the model. The models’ appropriateness was checked using different plots (QQ-plot, variance against fitted, variance against covariates, influence vs. cooks distance; plots not shown). Effects are reported as ratios (*R* = exp(*β*)) or differences (*β*), including the 95% confidence interval. As NSM values of LNFP-I and 2’FL were below the LoQ (≤15 mg/L and 53 mg/L, respectively) [33], associations involving LNFP-I and 2’FL were only modeled in the SM subgroup. *P*-values ≤0.05 were considered to be statistically significant. Because of the complex dependencies between the tested items, *p*-values were not adjusted for multiple testing. Essentially, we stress the fact that the results should be interpreted in terms of the occurring patterns instead of emphasizing single significant test results.

**Table 1:** Descriptive statistics of the cohort stratified by secretor status and overall given as median [Q1; Q3] for HMO and mean (standard deviation) for the other continuous variables. Group differences were tested using Kruskal-Wallis-tests (HMO) and t-tests (other variables)

	Secretor (*n* = 124)	Non-secretor (*n* = 21)	*p*-value	Total *n* = 145	n (non-missing)
Maternal age (years)	30.3 (4.12)	30.2 (4.95)	0.898	30.3 (4.24)	143
Pre-pregnancy BMI (kg/m^2^)	23.2 (3.78)	22.7 (3.83)	0.642	23.2 (3.78)	124
Gestational age (months)	40.0 (1.15)	40.2 (1.26)	0.494	40.0 (1.16)	145
Birth weight (g)	3467 (473)	3646 (486)	0.13	3493 (477)	145
Birth length (cm)	50.3 (2.37)	50.7 (2.28)	0.475	50.4 (2.36)	144
Birth head circumference (cm)	34.9 (1.51)	35.3 (1.33)	0.301	35.0 (1.48)	123
HMO (mg/L)					
2‘FL	2038 [1536;2722]	7.37 [6.66;10.0]	< 0.001	1931 [1317;2414]	145
3-FL	785 [531;1114]	2544 [2007;2889]	< 0.001	916 [565;1227]	145
3′SL	136 [119;162]	160 [134;173]	0.026	138 [120;166]	145
6′SL	151 [104;201]	139 [111;265]	0.39	150 [104;207]	145
LNT	547 [353;720]	699 [545;934]	0.01	567 [361;764]	145
LNnT	149 [117;200]	66.9 [38.8;89.9]	< 0.001	137 [90.8;185]	145
LNFP-I	473 [292;839]	2.00 [2.00;2.00]	< 0.001	416 [196;789]	145
LNFP-V	44.6 [31.3;61.9]	197 [141;235]	< 0.001	50.1 [33.7;83.5]	145
LNnFP	16.6 [9.51;22.7]	17.6 [7.56;21.6]	0.848	16.7 [8.82;22.7]	145

*BMI* Body Mass Index, *HMO* Human Milk Oligosaccharide, *2’FL* 2′-fucosyllactose, *3-FL* 3-fucosyllactose, *3′SL* 3′-sialyllactose, *6′SL* 6′-sialyllactose, *LNT* lacto-N-tetraose, *LNnT* lacto-N-neotetraose, *LNFP-I* lacto-N-fucopentaose-I, *LNFP-V* lacto-N-fucopentaose-V, *LNnFP* lacto-N-neofucopentaose

## Results

### Descriptive statistics and correlations between HMO

The cohort consists of 21 (14.7%) NSM and 122 (85.3%) SM. Two of the SM took part with 2 singleton pregnancies (Fig. 1). 2’FL, LNFP-I and LNnT concentrations were significantly lower in NSM, while 3-FL, 3′SL, LNT and LNFP-V concentrations were higher. Further descriptive statistics are given in Table 1.

**Fig. 2:**
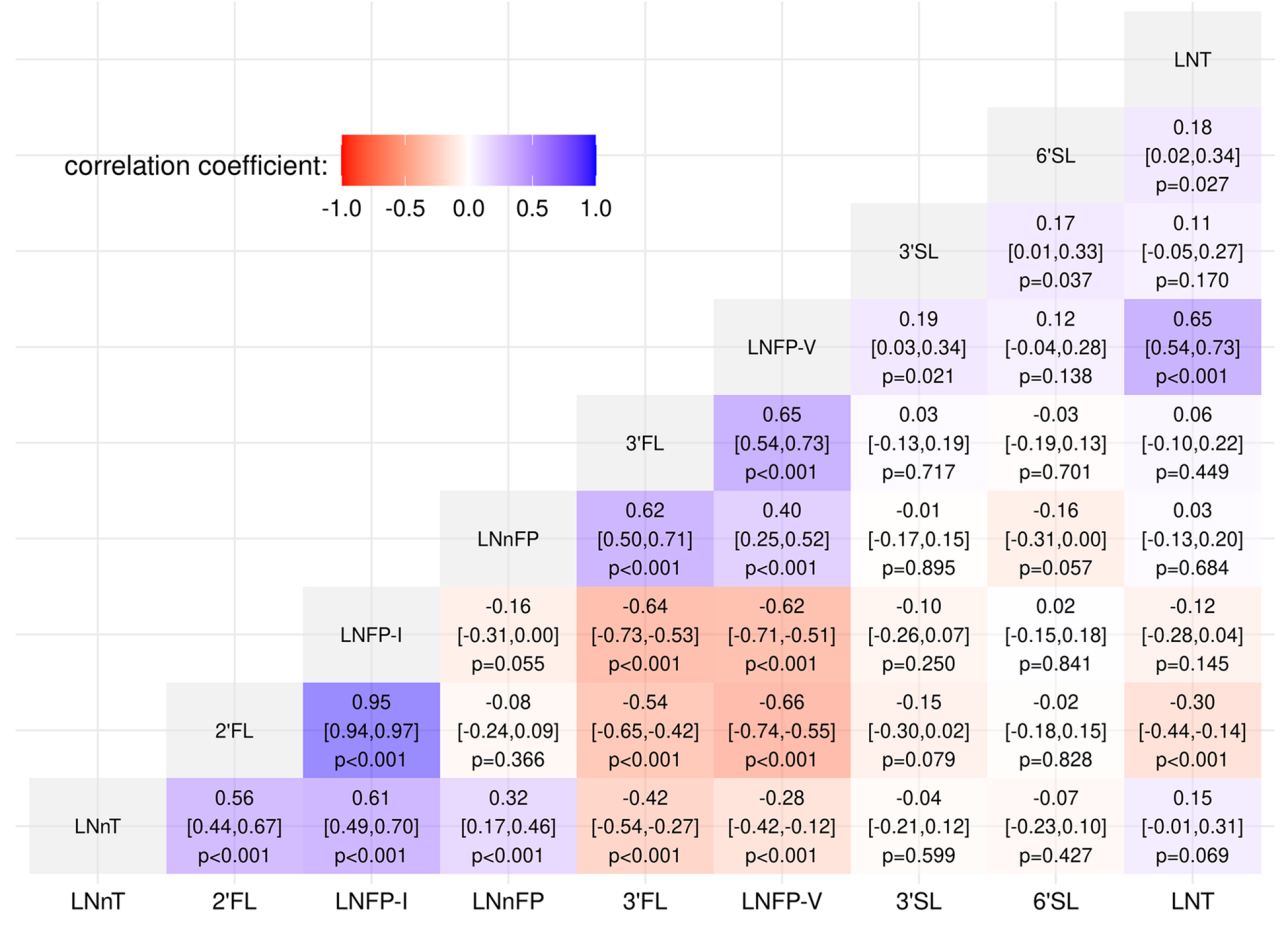
Correlogram of the log-transformed HMO values. Correlations coefficents, respective confidence intervals, and *p*-values are shown. HMO, Human Milk Oligosaccharide; 2’FL, 2′-fucosyllactose; 3-FL, 3-fucosyllactose; 3′SL, 3′-sialyllactose; 6′SL, 6′-sialyllactose; LNT, lacto-N-tetraose; LNnT, lacto-N-neotetraose; LNFP-I, lacto-N-fucopentaose-I; LNFP-V, lacto-N-fucopentaose-V; LNnFP, lacto-N-neofucopentaose

Correlation was highest between 2’FL and LNFP-I (*r* = 0.95, *p* < 0.001). Both were also positively correlated to LNnT (2’FL: *r* = 0.56, *p* < 0.001; LNFP-I: *r* = 0.61, *p* < 0.001) and negatively correlated to LNFP-V (2’FL: *r* = − 0.66, *p* < 0.001; LNFP-I: *r* = − 0.62, *p* < 0.001) and 3-FL (2’FL: *r* = − 0.54, *p* < 0.001; LNFP-I: *r* = − 0.64, *p* < 0.001) (Fig. 2).

### Maternal parameters, gestational age and birth parameters

The maternal age at birth was positively associated only with 3′SL (*R* = 1.06 [1.00,1.11] for every 5 years older, *p* = 0.03). Pre-pregnancy BMI was negatively associated with LNnT in NSM (*R* = 0.93 [0.90,0.97], *p* < 0.001); no association was found in SM. GA was positively associated with LNT (*R* = 1.08 [1.01,1.15], *p* = 0.03), 6′SL (*R* = 1.09 [1.03,1.16], *p* = 0.005) and LNFP-I (*R* = 1.15 [1.03,1.28], *p* = 0.012, only children from SM (SC)). LNFP-I (*R* = 1.2, [1.05,1.37], *p* = 0.008, only SC) and 3-FL (*R* = 0.92 [0.86,0.98], *p* = 0.011) were significantly associated with birth length (Supplementary Table S1).

### Height-SDS and growth velocity

**Fig. 3:**
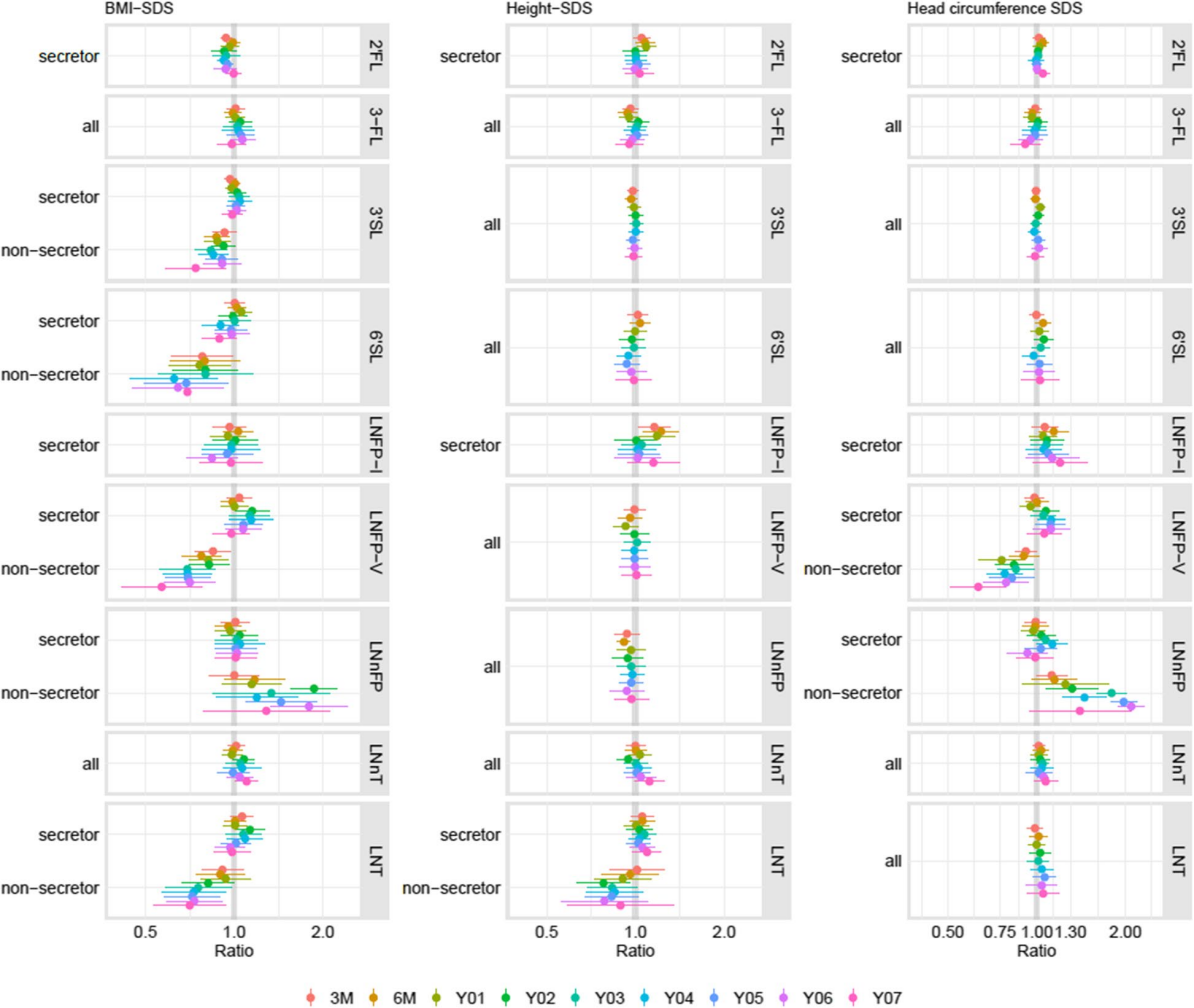
Associations between Human Milk Oligosaccharides at 3 months and BMI-, Height- and Head circumference SDS. They are presented for the different time points as ratio and 95% confidence interval. Ratios < 1 illustrate negative associations and ratios > 1 illustrate positive associations. If the effects differed between the secretor and the non-secretor group, both effects are given. Otherwise the overall effect is reported. BMI, Body Mass Index; SDS, Standard Deviation Score; 2’FL, 2′-fucosyllactose; 3-FL, 3-fucosyllactose; 3′SL, 3′-sialyllactose; 6′SL, 6′-sialyllactose; LNT, lacto-N-tetraose; LNnT, lacto-N-neotetraose; LNFP-I, lacto-N-fucopentaose-I; LNFP-V, lacto-N-fucopentaose-V; LNnFP, lacto-N-neofucopentaose; M, Months of Age; Y, Years of Age

The interaction between height-SDS and secretor status was significant for LNT. The association of height-SDS with LNT had a negative direction in children of NSM (NSC) (0.78 ≤ R ≤ 1.01) and a positive direction in SC (1.00 ≤ R ≤ 1.09; Supplementary Table S2; Fig. 3). However, the effects reached significance only for NSC at 2Y. LNFP-I was positively associated with height-SDS effects at 3 M, 6 M and 1Y (R ≈ 1.2, *p* ≤ 0.02); afterward, no further associations were found. Besides, there were consistently positive associations between height SDS and LNnT. However, statistical significance was only reached at 6Y. There was no evidence of associations between height-SDS and 2’FL, 3-FL, 3′SL, 6′SL, LNnFP, or LNFP-V.

The interaction between growth velocity and secretor status was significant for LNnT. In NSC, we found negative effects for 3 M–1Y (*R* = 0.95 [0.90,0.99], *p* = 0.01), 1Y–2Y (*R* = 0.80 [0.72,0.88], *p* < 0.001) and for 5Y–6Y (*R* = 0.71 [0.57,0.87], *p* = 0.002).

LNT and LNFP-V were negatively associated with growth velocity from 3 M–1Y (LNT: *R* = 0.97 [0.95,1.00], *p* = 0.02; LNFP-V: *R* = 0.97 [0.95,1.00], *p* = 0.04). LNFP-I and 3′SL were negatively associated with growth velocity from LNFP-I: 1Y–2Y (*R* = 0.90 [0.83,0.97] *p* = 0.008) and 3′SL: 4Y–5Y (*R* = 0.95 [0.90,1.00] *p* = 0.045). No significant associations were found for growth velocity and 2’FL, 3-FL, 6′SL and LNnFP (Supplementary Table S3).

### BMI-SDS

The interaction between BMI-SDS and secretor status was significant for 3′SL, 6′SL, LNT, LNFP-V and LNnFP. For NSC, we found consistently negative associations between BMI-SDS and 3′SL, 6′SL, LNT and LNFP-V at all time points (Supplementary Table S4; Fig. 3). However, statistical significance was reached only for LNT and LNFP-V at 2Y. Besides, we found consistently positive associations between BMI-SDS and LNnFP from 6 M onward. Statistical significance was reached at 02Y, 05Y and 06Y. We did not find evidence of associations between BMI-SDS and the HMO in SC when the models included the interaction term.

2’FL showed consistently negative associations with BMI-SDS (0.92 ≤ R ≤ 1; Supplementary Table S4; Fig. 3); statistical significance was reached at 3 M, 4Y and 5Y. The 3-FL was consistently positively associated with BMI-SDS between 3 M and 6Y; however, the results did not reach statistical significance. For LNnT and LNFP-I, no consistent patterns were found.

### Head circumference

The interaction between HC and secretor status was significant for LNFP-V and LNnFP. LNFP-V showed consistent, significantly negative associations with HC between 3 M and 7Y, with effect sizes varying between 0.63 and 0.92 in NSC (Supplementary Table S5; Fig. 3). SC had no notable pattern. LNnFP showed consistently positive effects on HC from 3 M–7Y, with effect sizes between 1.12 and 2.09 in NSC. In general, the effect sizes increased with age. Statistical significance was reached from 2Y–6Y. Again, we found no notable patterns in SC.

LNFP-I was consistently positively related to HC-SDS from 6 M–7Y with effect sizes between 1.02 and 1.20. However, most of the effects did not reach significance. There were no notable pattern or effects for 2’FL, 3-FL, 3′SL, 6′SL or LNT (Supplementary Table S5; Fig. 3).

### Comparisons in children of non-secretors vs. secretors

**Fig. 4:**
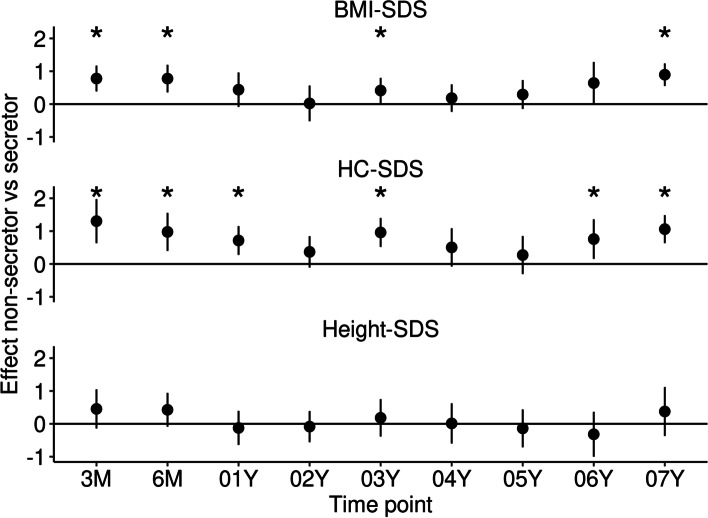
Mean differences in BMI-SDS, HC-SDS, and Height-SDS between the secretor and non-secretor group and 95%-confidence intervals. Mean SDS-differences > 0 represent higher values in non-secretors compared to secretors. From 3 months to 7 years of age BMI-SDS and HC-SDS were higher in non-secretors, especially at 3 M, 6 M, 01Y, and 03Y. BMI, Body Mass Index; HC, Head Circumference; SDS, Standard Deviation Score

NSC had a significantly higher BMI-SDS at 3 M (*β* = 0.8 [0.4,1.2], *p* < 0.001) and 6 M (*β* = 0.8 [0.4,1.2], *p* < 0.001). At 1 year, the direction of the association remained the same but the difference was not significant (Fig. 4).

At 3 M and 6 M, NSC tended to show an approximately + 0.5 higher height-SDS. However, there was no statistically significant effect. HC-SDS was astoundingly higher in NSC at 3 M (*β =* 1.3 [0.6,2.0], *p* < 0.001), 6 M (*β* = 1.0 [0.4,1.6], *p* = 0.001), and 1Y (*β* = 0.7 [0.3,1.2], *p* = 0.002). Even afterwards, HC-SDS stayed higher in NSC with effect sizes between *β* = 0.4 and *β* = 1.0, reaching significance at 3Y, 6Y, and 7Y (Fig. 4).

## Discussion

Our study aimed to investigate how HMO are associated with infant anthropometry from 3 months to 7 years of age and to identify any consistent patterns that could signify an important role of HMO for early growth. We found a consistent inverse association of growth velocity with LNnT only in NSC group. In addition, NSC had consistently higher BMI than SC. We also explored the influence of maternal and infant factors in HMO composition with maternal age, gestational age and pre-pregnancy BMI significantly associated with some of the HMO.

Recent studies proposed that HMO, besides their antimicrobial effects, may be involved in infant growth and development. HMO are indigestible but can be fermented, at least partially, by the infant’s microbiome [16–18, 20, 40–42]. This promotes the growth and activity of commensal bacteria such as *Bifidobacterium* and *Bacteroides spp.* and supports the gastrointestinal tract’s maturation and the immune system [43]. HMO may also reduce the risk of infections by protecting against colonization with pathogenic microorganisms. It is proposed that they can act as decoys, inhibiting the pathogen anchoring to the human epithelial cells [21, 22, 44, 45].

It was recently suggested that sialylated oligosaccharides may exert a microbiota-dependent promotion of anabolic function in animal models by increasing the nutrient’s efficiency, promoting better growth and physical development [46, 47]. Given the HMO-microbiome interaction and the microbiome’s proposed effect on nutrient efficiency, combined with maternal factors as pre-pregnancy BMI, the HMO composition of breast milk may also affect infant growth. Previous studies [9, 10, 12–14] investigating associations between HMO and infant growth obtained conflicting results. Alderete et al. identified associations between LNFP-I and lower infantile weight, but not with pre-pregnancy BMI [10]. In contrast, more recent studies found 2’FL positively associated with both child growth and pre-pregnancy BMI [12, 13]. Other studies reported no associations between HMO composition or secretor status with child growth [9, 14].

Despite the variability in results, 2 recent studies indicated a role of sialylated HMO in infant growth considering maternal BMI [14, 48]. Binia et al. found moderate associations between HMO and infant growth and body composition during the first 4 months of life in a cohort of predominantly healthy babies and mothers with normal BMI. They reported significant associations of higher growth rate during the 4 months of lactation with higher 3′SL, expressed as Area Under the Curve of HMO concentrations at all visits, a potentially better measure of HMO exposure. Saben et al. confirmed the positive association of several sialylated HMO, including 3′SL but also total acidic HMO with infant growth during the first 6 months of life, including also mothers with obesity. The growth and body composition of the healthy infants were independent of maternal pre-pregnancy BMI. Interestingly, Saben et al. used calculated milk and HMO intake and not only concentrations, an attempt again to better quantify exposure to HMO. The study was limited to 1 single time-point of HMO quantification at 2 months. Both studies lacked the longer follow-up of infant growth, which could be a better indicator of future risk to obesity. Finally, neither of these 2 studies confirmed the previous observations by Lagström et al. [12] and Larsson et al. [13] on the positive association of 2’FL and the negative association of LNnT with infant growth. Another rare example of HMO intake being quantitatively measured looked at HMO intake at several time points up to 12 months. However, the total (not individual) HMO intake was calculated. They found higher HMO concentrations associated with higher percentages of the fat-free mass at 2, 5, 9, and 12 months of age, whereas fat mass was negatively related to higher HMO intake at 5, 9, and 12 months of age [49].

Despite measuring HMO only at 3 months postpartum from only 20 mL of milk and no full breast expression, limiting insights on associations between growth and the changing HMO exposure over time, we could include growth data from birth until 7 years of age in a relatively strong sample size for this long follow-up compared to other studies. Indeed, we found higher BMI and HC SDS in NSC than SC. Although significance was not achieved at all time points, the probability of only positive results is *p* < 0.002. Growth velocity but not BMI was inversely correlated with LNnT at 3 M–1Y and 1Y–2Y in NSC, supporting the findings from Lagström et al. [12]. Regarding the association between infant growth and sialylated HMO 3′SL and 6′SL, we found a negative association with BMI-SDS in NSC but not with growth velocity, as previously reported [14, 48]. Our study is an exploratory approach to identify associations between maternal, infant parameters and HMO. Our findings could be affected by false positive results, however the associations of growth velocity with LNnT and BMI with 2’FL are consistent for multiple time-points. We did not have milk intake measurements available in our study; future studies need to include these parameters to estimate more accurately the exposure of the infant gastrointestinal tract to HMO.

Included mothers had a mean BMI of 23.2 (3.78) kg/m^2^ and a mean age of 30 years, similar to other study populations [14, 48]. In line with previous results, we found a negative association between pre-pregnancy BMI and LNnT in NSM [12]. However, other studies reported a positive or no association [27, 28, 48]. This highlights the variability of reported HMO associations, reflecting possible differences in methods or non-measured confounders. Therefore, future studies examining the role of HMO in growth and metabolic health should consider maternal physiology and other human milk components, such as proteins and lipids.

One in vivo intervention study with sialylated oligosaccharides [47] did report growth recovery following treatment with sialylated oligosaccharides in animal models of undernutrition. Recent randomized placebo controlled clinical trials testing infant formula containing specific individual HMO (2’FL or 2’FL and LNnT) effect on growth showed no differences in infant growth up to 12 months of age [50, 51]. Their results may imply that the impact of single or a few HMO may not have a large effect in the growth of healthy infants. In addition, these trials were randomized, whereas observational studies are not and factors other than HMO may confound associations. Ideally, future intervention studies with HMO mixes closer to those in human milk should be followed beyond the first year of life. Observational studies like the present could highlight the importance of maternal and infant characteristics in the relationship between HMO and infant growth and development. The conflicting reported results however from recent observational data call for hypothesis-driven studies with detailed meta-data collection to test the specific role of groups rather than single HMO in influencing early growth and composition.

## Conclusion

Our results suggest that associations between HMO and infant growth may extend beyond the breastfeeding period and interventional studies are needed to elucidate their influence on infant weight, height and body composition. Our study also confirms the value of long-term follow-up of breastfed infants and the inclusion of both maternal and infant factors to understand the role of HMO in growth and development [27, 29].

## Supplementary Information

**Additional file 1: Supplementary Table S1.** Associations between Human Milk Oligosaccharides at 3 months and maternal and birth parameter are presented as ratio, 95% confidence interval, and *p*-value. If the effects differed between the secretor and the non-secretor group, both effects are given. Otherwise the overall effect is reported. **Supplementary Table S2.** Associations between Human Milk Oligosaccharides at 3 months and height-SDS at the different time points are presented as ratio, 95% confidence interval, and *p*-value. If the effects differed between the secretor and the non-secretor group, both effects are given. Otherwise the overall effect is reported. **Supplementary Table S3.** Associations between Human Milk Oligosaccharides at 3 months and growth velocity at the different time points are presented as ratio, 95% confidence interval, and *p*-value. If the effects differed between the secretor and the non-secretor group, both effects are given. Otherwise the overall effect is reported. **Supplementary Table S4.** Associations between Human Milk Oligosaccharides at 3 months and BMI-SDS at the different time points are presented as ratio, 95% confidence interval, and p-value. If the effects differed between the secretor and the non-secretor group, both effects are given. Otherwise the overall effect is reported. **Supplementary Table S5.** Associations between Human Milk Oligosaccharides at 3 months and Head Circumference SDS at the different time points are presented as ratio, 95% confidence interval, and p-value. If the effects differed between the secretor and the non-secretor group, both effects are given. Otherwise the overall effect is reported.

## Data Availability

The legal requirements and the given informed consent do not allow public sharing of the dataset. Interested researchers can contact the research data management of the Medical Faculty, University Leipzig: rdm@medizin.uni-leipzig.de for further information. The dataset ID is PV450.

## Abbreviations

HMO: Human Milk Oligosaccharide
2’FL: 2′-fucosyllactose
3-FL: 3-fucosyllactose
3′SL: 3′-sialyllactose
6′SL: 6′-sialyllactose
LNT: Lacto-N-tetraose
LNnT: Lacto-N-neotetraose
LNFP-I: Lacto-N-fucopentaose-I
LNFP-V: Lacto-N-fucopentaose-V
LNnFP: Lacto-N-neofucopentaose
SM: Mothers who are Secretors
NSM: Mothers who are Non-Secretors
SC: Children of Mothers who are Secretors
NSC: Children of Mothers who are Non-Secretors
BMI: Body Mass Index
HC: Head Circumference
GA: Gestational Age
SDS: Standard Deviation Score
Glc: Glucose
GlcNAc: N-Acetylglucosamine
Gal: Galactose
Fuc: Fucose
Neu5Ac: N-Acetylneuraminic acid
LoQ: Limit of Quantification
