# Supplementary material for: Concentrations of oligosaccharides in human milk and child growth
Source: BMC Pediatr. 2021 Oct 30;21:481. doi: 10.1186/s12887-021-02953-0 (PMC8556924; doi:10.1186/s12887-021-02953-0)
Supplement: Supplementary file 1 — Additional file 1: Supplementary Table S1. Associations between Human Milk Oligosaccharides at 3 months and maternal and birth parameter are presented as ratio, 95% confidence interval, and p-value. If the effects differed between the secretor and the non-secretor group, both effects are given. Otherwise the overall effect is reported. Supplementary Table S2. Associations between Human Milk Oligosaccharides at 3 months and height-SDS at the different time points are presented as ratio, 95% confidence interval, and p-value. If the effects differed between the secretor and the non-secretor group, both effects are given. Otherwise the overall effect is reported. Supplementary Table S3. Associations between Human Milk Oligosaccharides at 3 months and growth velocity at the different time points are presented as ratio, 95% confidence interval, and p-value. If the effects differed between the secretor and the non-secretor group, both effects are given. Otherwise the overall effect is reported. Supplementary Table S4. Associations between Human Milk Oligosaccharides at 3 months and BMI-SDS at the different time points are presented as ratio, 95% confidence interval, and p-value. If the effects differed between the secretor and the non-secretor group, both effects are given. Otherwise the overall effect is reported. Supplementary Table S5. Associations between Human Milk Oligosaccharides at 3 months and Head Circumference SDS at the different time points are presented as ratio, 95% confidence interval, and p-value. If the effects differed between the secretor and the non-secretor group, both effects are given. Otherwise the overall effect is reported. [file 12887_2021_2953_MOESM1_ESM.docx]

**Concentrations of oligosaccharides in human milk and child growth**

Philipp Menzel, Mandy Vogel, Dr., Sean Austin, Norbert Sprenger, Dr., Nico Grafe, Cornelia Hilbert, Dr., Anne Jurkutat,

Wieland Kiess, Prof., and Aristea Binia, Dr.

Abbreviations for all Supplementary Tables S1 – S5

*BMI, body mass index; SDS, standard deviation score; se-, non-secretors; se+, secretors; 2'FL, 2'-Fucosyllactose; 3-FL, 3-Fucosyllactose; 3'SL, 3'-Sialyllactose; 6'SL, 6'-Sialyllactose; LNT, Lacto-N-Tetraose; LNnT, Lacto-N-Neotetraose; LNFP-I, Lacto-N-Fucopentaose I; LNFP-V, Lacto-N-Fucopentaose V; LNnFP, Lacto-N-Neofucopentaose; M, months of age; Y, years of age.*

Supplementary Table S1 - Associations between Human Milk Oligosaccharides at 3 months and maternal and birth parameter are presented as ratio, 95% confidence interval, and p-value. If the effects differed between the secretor and the non-secretor group, both effects are given. Otherwise the overall effect is reported.

| Covariate | 2'FL | 3-FL | 3'SL | 6'SL | LNT | LNnT | | LNFP-I | LNFP-V | LNnFP |
| --- | --- | --- | --- | --- | --- | --- | --- | --- | --- | --- |
|  | se+ |  |  |  |  | se- | se+ | se+ |  |  |
| Maternal Age (+5 years) | R=0.95 [0.88,1.02] p=0.141 | R=1.05 [0.96,1.15] p=0.314 | R=1.06 [1.00,1.11] p=0.034 | R=0.99 [0.90,1.08] p=0.788 | R=0.99 [0.90,1.09] p=0.792 | R=0.99 [0.91,1.08] p=0.810 | | R=0.91 [0.78,1.06] p=0.233 | R=1.05 [0.95,1.16] p=0.343 | R=0.99 [0.88,1.11] p=0.881 |
| pre-pregnancy BMI | R=1.01 [0.99,1.03] p=0.317 | R=1.01 [0.99,1.03] p=0.254 | R=1.00 [0.99,1.01] p=0.912 | R=1.01 [0.99,1.03] p=0.215 | R=0.99 [0.96,1.01] p=0.250 | R=0.93 [0.90,0.97] p<0.001 | R=1.01 [0.99,1.03] p=0.383 | R=0.99 [0.96,1.03] p=0.760 | R=0.99 [0.97,1.02] p=0.652 | R=1.00 [0.97,1.03] p=0.828 |
| Gestational Age (+1 week) | R=1.02 [0.97,1.07] p=0.516 | R=0.94 [0.87,1.00] p=0.060 | R=0.99 [0.95,1.03] p=0.534 | R=1.09 [1.03,1.16] p=0.005 | R=1.08 [1.01,1.15] p=0.033 | R=0.98 [0.93,1.04] p=0.580 | | R=1.15 [1.03,1.28] p=0.012 | R=1.04 [0.97,1.12] p=0.277 | R=0.99 [0.91,1.08] p=0.900 |
| Birth Length SDS | R=1.06 [0.99,1.14] p=0.086 | R=0.92 [0.86,0.98] p=0.011 | R=0.99 [0.94,1.04] p=0.553 | R=1.03 [0.96,1.12] p=0.401 | R=1.04 [0.95,1.13] p=0.397 | R=1.04 [0.96,1.13] p=0.318 | | R=1.20 [1.05,1.37] p=0.008 | R=0.99 [0.90,1.08] p=0.764 | R=0.93 [0.84,1.03] p=0.173 |
| Birth Weight SDS | R=1.01 [0.94,1.09] p=0.728 | R=0.98 [0.92,1.05] p=0.619 | R=1.00 [0.95,1.05] p=0.980 | R=1.04 [0.96,1.13] p=0.290 | R=1.06 [0.97,1.16] p=0.202 | R=1.01 [0.93,1.09] p=0.858 | | R=1.13 [0.99,1.29] p=0.074 | R=1.03 [0.94,1.13] p=0.558 | R=0.96 [0.86,1.07] p=0.493 |

Supplementary Table S2 - Associations between Human Milk Oligosaccharides at 3 months and height-SDS at the different time points are presented as ratio, 95% confidence interval, and p-value. If the effects differed between the secretor and the non-secretor group, both effects are given. Otherwise the overall effect is reported.

| Time point | 2'FL | 3-FL | 3'SL | 6'SL | LNT | | LNnT | LNFP-I | LNFP-V | LNnFP |
| --- | --- | --- | --- | --- | --- | --- | --- | --- | --- | --- |
|  | se+ |  |  |  | se- | se+ |  | se+ |  |  |
| 3M | R=1.05 [0.98,1.12] p=0.167 | R=0.96 [0.90,1.02] p=0.218 | R=0.98 [0.94,1.02] p=0.348 | R=1.02 [0.94,1.10] p=0.631 | R=1.01 [0.81,1.26] p=0.911 | R=1.05 [0.96,1.15] p=0.251 | R=1.00 [0.93,1.08] p=0.976 | R=1.16 [1.02,1.32] p=0.024 | R=0.99 [0.91,1.08] p=0.848 | R=0.94 [0.85,1.04] p=0.192 |
| 6M | R=1.08 [1.00,1.16] p=0.043 | R=0.94 [0.87,1.01] p=0.091 | R=0.97 [0.92,1.01] p=0.164 | R=1.04 [0.95,1.13] p=0.384 | R=0.96 [0.77,1.20] p=0.713 | R=1.06 [0.96,1.16] p=0.262 | R=1.00 [0.92,1.09] p=0.939 | R=1.22 [1.06,1.41] p=0.005 | R=0.96 [0.87,1.05] p=0.373 | R=0.91 [0.87,0.96] p<0.001 |
| 1Y | R=1.09 [1.01,1.17] p=0.029 | R=0.95 [0.88,1.03] p=0.201 | R=0.99 [0.94,1.04] p=0.622 | R=1.00 [0.91,1.09] p=0.951 | R=0.91 [0.72,1.14] p=0.385 | R=1.00 [0.91,1.11] p=0.925 | R=1.04 [0.95,1.13] p=0.416 | R=1.18 [1.03,1.36] p=0.019 | R=0.93 [0.84,1.02] p=0.116 | R=0.97 [0.86,1.08] p=0.548 |
| 2Y | R=1.00 [0.91,1.10] p=0.980 | R=1.02 [0.94,1.11] p=0.613 | R=1.00 [0.95,1.06] p=0.992 | R=0.97 [0.88,1.07] p=0.574 | R=0.78 [0.63,0.96] p=0.019 | R=1.03 [0.93,1.14] p=0.539 | R=0.94 [0.87,1.03] p=0.184 | R=1.01 [0.85,1.19] p=0.941 | R=0.99 [0.89,1.11] p=0.883 | R=0.94 [0.83,1.07] p=0.331 |
| 3Y | R=1.00 [0.92,1.09] p=0.950 | R=1.01 [0.94,1.09] p=0.788 | R=1.01 [0.95,1.06] p=0.834 | R=0.99 [0.90,1.09] p=0.790 | R=0.83 [0.68,1.02] p=0.072 | R=1.07 [0.98,1.18] p=0.145 | R=1.00 [0.91,1.10] p=0.927 | R=1.05 [0.90,1.22] p=0.534 | R=1.01 [0.91,1.12] p=0.812 | R=0.97 [0.86,1.09] p=0.563 |
| 4Y | R=1.00 [0.92,1.10] p=0.927 | R=1.00 [0.92,1.08] p=0.913 | R=1.00 [0.95,1.06] p=0.949 | R=0.95 [0.86,1.04] p=0.248 | R=0.85 [0.68,1.07] p=0.159 | R=1.03 [0.95,1.12] p=0.483 | R=1.03 [0.93,1.13] p=0.616 | R=1.01 [0.87,1.18] p=0.844 | R=0.99 [0.90,1.10] p=0.852 | R=0.98 [0.89,1.08] p=0.620 |
| 5Y | R=1.02 [0.93,1.12] p=0.620 | R=1.01 [0.93,1.11] p=0.793 | R=0.98 [0.93,1.03] p=0.412 | R=0.93 [0.85,1.03] p=0.168 | R=0.83 [0.67,1.03] p=0.086 | R=1.02 [0.93,1.12] p=0.628 | R=1.01 [0.91,1.12] p=0.850 | R=1.03 [0.87,1.21] p=0.729 | R=0.99 [0.89,1.10] p=0.897 | R=0.97 [0.88,1.06] p=0.473 |
| 6Y | R=1.00 [0.90,1.10] p=0.926 | R=0.97 [0.89,1.07] p=0.593 | R=0.99 [0.94,1.05] p=0.794 | R=0.97 [0.86,1.09] p=0.581 | R=0.78 [0.55,1.11] p=0.167 | R=1.06 [0.96,1.17] p=0.274 | R=1.04 [0.93,1.17] p=0.471 | R=1.02 [0.84,1.23] p=0.858 | R=1.00 [0.88,1.12] p=0.944 | R=0.93 [0.82,1.07] p=0.319 |
| 7Y | R=1.03 [0.92,1.15] p=0.558 | R=0.95 [0.86,1.06] p=0.361 | R=0.98 [0.92,1.05] p=0.622 | R=0.99 [0.86,1.14] p=0.861 | R=0.89 [0.58,1.36] p=0.586 | R=1.09 [0.98,1.23] p=0.122 | R=1.11 [0.99,1.25] p=0.065 | R=1.15 [0.93,1.42] p=0.193 | R=1.01 [0.89,1.14] p=0.894 | R=0.97 [0.85,1.11] p=0.639 |

Supplementary Table S3 - Associations between Human Milk Oligosaccharides at 3 months and growth velocity at the different time points are presented as ratio, 95% confidence interval, and p-value. If the effects differed between the secretor and the non-secretor group, both effects are given. Otherwise the overall effect is reported.

| Time point | 2'FL | 3-FL | 3'SL | 6'SL | LNT | LNnT | | LNFP-I | LNFP-V | LNnFP |
| --- | --- | --- | --- | --- | --- | --- | --- | --- | --- | --- |
|  | se+ |  |  |  |  | se- | se+ | se+ |  |  |
| 3M-1Y | R=1.01 [0.99,1.03] p=0.483 | R=1.01 [0.98,1.03] p=0.635 | R=1.00 [0.98,1.01] p=0.881 | R=0.99 [0.96,1.01] p=0.276 | R=0.97 [0.95,1.00] p=0.022 | R=0.95 [0.90,0.99] p=0.014 | R=1.01 [0.98,1.04] p=0.538 | R=0.99 [0.95,1.03] p=0.528 | R=0.97 [0.95,1.00] p=0.041 | R=1.02 [0.98,1.06] p=0.282 |
| 1Y-2Y | R=0.96 [0.91,1.00] p=0.073 | R=1.04 [1.00,1.10] p=0.073 | R=1.00 [0.97,1.03] p=0.853 | R=0.98 [0.93,1.03] p=0.364 | R=0.98 [0.93,1.04] p=0.503 | R=0.80 [0.72,0.88] p<0.001 | R=0.95 [0.89,1.01] p=0.092 | R=0.90 [0.83,0.97] p=0.008 | R=1.04 [0.98,1.10] p=0.244 | R=1.01 [0.94,1.08] p=0.757 |
| 2Y-3Y | R=1.01 [0.95,1.07] p=0.776 | R=1.01 [0.96,1.06] p=0.804 | R=1.01 [0.98,1.04] p=0.514 | R=1.00 [0.95,1.07] p=0.887 | R=1.04 [0.98,1.11] p=0.210 | R=1.01 [0.90,1.13] p=0.855 | R=1.10 [1.02,1.18] p=0.009 | R=1.05 [0.95,1.17] p=0.339 | R=1.01 [0.94,1.07] p=0.830 | R=1.04 [0.96,1.12] p=0.297 |
| 3Y-4Y | R=0.98 [0.91,1.05] p=0.558 | R=1.02 [0.95,1.10] p=0.507 | R=1.02 [0.97,1.07] p=0.378 | R=0.99 [0.91,1.07] p=0.748 | R=0.98 [0.92,1.05] p=0.598 | R=0.90 [0.73,1.11] p=0.324 | R=1.01 [0.93,1.11] p=0.785 | R=0.96 [0.86,1.08] p=0.538 | R=1.02 [0.94,1.11] p=0.656 | R=0.99 [0.90,1.09] p=0.860 |
| 4Y-5Y | R=0.99 [0.90,1.09] p=0.878 | R=0.98 [0.88,1.09] p=0.697 | R=0.95 [0.90,1.00] p=0.045 | R=0.91 [0.82,1.01] p=0.068 | R=1.00 [0.91,1.10] p=0.970 | R=0.93 [0.70,1.24] p=0.629 | R=0.98 [0.87,1.11] p=0.780 | R=1.02 [0.86,1.21] p=0.817 | R=0.96 [0.85,1.07] p=0.443 | R=0.90 [0.79,1.02] p=0.109 |
| 5Y-6Y | R=0.98 [0.87,1.11] p=0.774 | R=0.94 [0.85,1.04] p=0.212 | R=1.01 [0.94,1.08] p=0.876 | R=1.06 [0.92,1.22] p=0.431 | R=1.08 [0.95,1.22] p=0.260 | R=0.71 [0.57,0.87] p=0.002 | R=1.00 [0.86,1.17] p=0.981 | R=1.09 [0.87,1.36] p=0.462 | R=1.02 [0.90,1.17] p=0.715 | R=0.90 [0.77,1.06] p=0.206 |
| 6Y-7Y | R=1.00 [0.81,1.24] p=0.994 | R=1.02 [0.87,1.19] p=0.786 | R=1.00 [0.88,1.13] p=0.981 | R=1.00 [0.78,1.27] p=0.999 | R=0.97 [0.77,1.22] p=0.786 | R=1.05 [0.64,1.74] p=0.843 | R=1.12 [0.88,1.42] p=0.345 | R=1.03 [0.68,1.56] p=0.878 | R=0.94 [0.76,1.18] p=0.600 | R=1.13 [0.88,1.46] p=0.323 |

Supplementary Table S4 (Part 1/2) - Associations between Human Milk Oligosaccharides at 3 months and BMI-SDS at the different time points are presented as ratio, 95% confidence interval, and p-value. If the effects differed between the secretor and the non-secretor group, both effects are given. Otherwise the overall effect is reported.

| Time point | 2'FL | 3-FL | 3'SL | | 6'SL | | LNT | | LNnT | LNFP-I |
| --- | --- | --- | --- | --- | --- | --- | --- | --- | --- | --- |
|  |  |  | Se- | Se+ | Se- | Se+ | Se- | Se+ |  | Se+ |
| 3M | R=0.94 [0.90,0.98] p=0.003 | R=1.01 [0.94,1.08] p=0.759 | R=0.93 [0.85,1.02] p=0.105 | R=0.97 [0.92,1.01] p=0.167 | R=0.78 [0.61,0.99] p=0.041 | R=1.00 [0.93,1.09] p=0.911 | R=0.91 [0.77,1.08] p=0.270 | R=1.06 [0.97,1.16] p=0.171 | R=1.02 [0.95,1.09] p=0.658 | R=0.96 [0.85,1.10] p=0.578 |
| 6M | R=0.99 [0.94,1.04] p=0.691 | R=0.99 [0.92,1.06] p=0.752 | R=0.87 [0.79,0.96] p=0.006 | R=1.01 [0.96,1.05] p=0.789 | R=0.79 [0.60,1.05] p=0.098 | R=1.02 [0.95,1.10] p=0.557 | R=0.90 [0.74,1.09] p=0.259 | R=1.01 [0.93,1.09] p=0.861 | R=0.99 [0.92,1.06] p=0.787 | R=1.03 [0.91,1.17] p=0.603 |
| 1Y | R=0.96 [0.90,1.03] p=0.301 | R=1.01 [0.93,1.09] p=0.827 | R=0.88 [0.79,0.97] p=0.013 | R=0.98 [0.93,1.03] p=0.421 | R=0.76 [0.60,0.98] p=0.030 | R=1.06 [0.97,1.15] p=0.185 | R=0.94 [0.77,1.14] p=0.502 | R=1.01 [0.92,1.11] p=0.879 | R=0.98 [0.91,1.06] p=0.649 | R=0.95 [0.83,1.09] p=0.479 |
| 2Y | R=0.92 [0.84,1.02] p=0.114 | R=1.05 [0.96,1.15] p=0.286 | R=0.92 [0.84,1.01] p=0.080 | R=1.02 [0.96,1.10] p=0.485 | R=0.80 [0.62,1.04] p=0.088 | R=0.99 [0.89,1.11] p=0.870 | R=0.82 [0.67,1.00] p=0.050 | R=1.13 [1.01,1.27] p=0.037 | R=1.08 [0.99,1.17] p=0.077 | R=1.01 [0.84,1.21] p=0.925 |
| 3Y | R=0.94 [0.84,1.05] p=0.255 | R=1.02 [0.92,1.15] p=0.664 | R=0.83 [0.73,0.94] p=0.004 | R=1.04 [0.96,1.12] p=0.313 | R=0.80 [0.55,1.16] p=0.235 | R=1.00 [0.88,1.14] p=0.952 | R=0.75 [0.58,0.98] p=0.035 | R=1.07 [0.93,1.23] p=0.319 | R=1.05 [0.94,1.17] p=0.404 | R=0.98 [0.79,1.21] p=0.831 |
| 4Y | R=0.92 [0.88,0.96] p<0.001 | R=1.03 [0.91,1.17] p=0.605 | R=0.85 [0.76,0.96] p=0.007 | R=1.05 [0.95,1.15] p=0.348 | R=0.63 [0.44,0.89] p=0.009 | R=0.90 [0.78,1.04] p=0.153 | R=0.73 [0.57,0.94] p=0.014 | R=1.09 [0.95,1.26] p=0.230 | R=1.06 [0.92,1.23] p=0.405 | R=0.98 [0.78,1.23] p=0.850 |
| 5Y | R=0.95 [0.91,0.99] p=0.019 | R=1.05 [0.95,1.18] p=0.327 | R=0.91 [0.80,1.03] p=0.134 | R=1.02 [0.95,1.09] p=0.665 | R=0.69 [0.49,0.96] p=0.027 | R=0.98 [0.86,1.10] p=0.693 | R=0.72 [0.58,0.90] p=0.004 | R=1.01 [0.90,1.14] p=0.803 | R=0.99 [0.87,1.12] p=0.884 | R=0.95 [0.77,1.16] p=0.589 |
| 6Y | R=0.94 [0.86,1.02] p=0.137 | R=1.07 [0.97,1.18] p=0.203 | R=0.91 [0.78,1.06] p=0.225 | R=1.02 [0.95,1.09] p=0.565 | R=0.64 [0.45,0.93] p=0.019 | R=0.98 [0.86,1.12] p=0.778 | R=0.73 [0.59,0.91] p=0.007 | R=0.97 [0.86,1.09] p=0.596 | R=1.04 [0.94,1.15] p=0.397 | R=0.84 [0.69,1.03] p=0.096 |
| 7Y | R=1.00 [0.93,1.06] p=0.891 | R=0.98 [0.87,1.10] p=0.749 | R=0.74 [0.58,0.94] p=0.019 | R=0.98 [0.91,1.06] p=0.688 | R=0.69 [0.37,1.30] p=0.253 | R=0.89 [0.78,1.02] p=0.104 | R=0.71 [0.53,0.94] p=0.022 | R=0.98 [0.85,1.14] p=0.818 | R=1.10 [1.01,1.20] p=0.032 | R=0.97 [0.76,1.25] p=0.836 |

Table continued: Supplementary Table S4 (Part 2/2)

| Time point | LNFP-V | | LNnFP | |
| --- | --- | --- | --- | --- |
|  | Se- | Se+ | Se- | Se+ |
| 3M | R=0.85 [0.74,0.98] p=0.020 | R=1.04 [0.94,1.15] p=0.414 | R=1.00 [0.82,1.22] p=0.991 | R=1.01 [0.90,1.13] p=0.858 |
| 6M | R=0.77 [0.66,0.90] p=0.001 | R=0.99 [0.90,1.08] p=0.761 | R=1.17 [0.92,1.49] p=0.190 | R=0.95 [0.86,1.06] p=0.380 |
| 1Y | R=0.82 [0.70,0.96] p=0.011 | R=1.00 [0.90,1.12] p=0.961 | R=1.15 [0.90,1.45] p=0.252 | R=0.97 [0.86,1.10] p=0.610 |
| 2Y | R=0.82 [0.70,0.97] p=0.017 | R=1.15 [1.00,1.32] p=0.045 | R=1.87 [1.56,2.24] p<0.001 | R=1.04 [0.90,1.21] p=0.556 |
| 3Y | R=0.69 [0.55,0.87] p=0.001 | R=1.13 [0.96,1.32] p=0.136 | R=1.34 [0.84,2.13] p=0.218 | R=1.02 [0.86,1.21] p=0.823 |
| 4Y | R=0.69 [0.57,0.84] p<0.001 | R=1.14 [0.96,1.36] p=0.138 | R=1.19 [0.86,1.65] p=0.283 | R=1.05 [0.86,1.28] p=0.647 |
| 5Y | R=0.70 [0.58,0.83] p<0.001 | R=1.07 [0.92,1.25] p=0.345 | R=1.44 [1.08,1.92] p=0.012 | R=1.01 [0.86,1.19] p=0.891 |
| 6Y | R=0.71 [0.58,0.86] p=0.001 | R=1.07 [0.93,1.24] p=0.331 | R=1.79 [1.32,2.44] p<0.001 | R=1.02 [0.87,1.21] p=0.784 |
| 7Y | R=0.57 [0.41,0.78] p=0.001 | R=0.98 [0.84,1.13] p=0.759 | R=1.28 [0.78,2.12] p=0.326 | R=1.01 [0.86,1.19] p=0.884 |

Supplementary Table S5 - Associations between Human Milk Oligosaccharides at 3 months and Head Circumference SDS at the different time points are presented as ratio, 95% confidence interval, and p-value. If the effects differed between the secretor and the non-secretor group, both effects are given. Otherwise the overall effect is reported.

| Time point | 2'FL | 3-FL | 3'SL | 6'SL | LNT | LNnT | LNFP-I | LNFP-V | | LNnFP | |
| --- | --- | --- | --- | --- | --- | --- | --- | --- | --- | --- | --- |
|  | se+ |  |  |  |  |  | se+ | se- | se+ | se- | se+ |
| 3M | R=1.02 [0.97,1.07] p=0.510 | R=0.99 [0.94,1.04] p=0.628 | R=0.99 [0.96,1.03] p=0.705 | R=1.00 [0.94,1.05] p=0.906 | R=0.98 [0.93,1.04] p=0.601 | R=1.02 [0.97,1.06] p=0.530 | R=1.06 [0.96,1.18] p=0.228 | R=0.92 [0.84,1.00] p=0.043 | R=0.98 [0.91,1.06] p=0.628 | R=1.12 [1.00,1.27] p=0.057 | R=0.99 [0.91,1.08] p=0.834 |
| 6M | R=1.05 [1.00,1.10] p=0.051 | R=0.97 [0.91,1.03] p=0.270 | R=0.99 [0.95,1.03] p=0.610 | R=1.05 [0.99,1.12] p=0.119 | R=1.01 [0.94,1.09] p=0.687 | R=1.03 [0.97,1.10] p=0.267 | R=1.14 [1.01,1.29] p=0.028 | R=0.90 [0.80,1.02] p=0.090 | R=1.00 [0.92,1.09] p=0.982 | R=1.15 [0.96,1.37] p=0.124 | R=0.99 [0.89,1.10] p=0.814 |
| 1Y | R=1.02 [0.97,1.08] p=0.448 | R=0.96 [0.90,1.03] p=0.255 | R=1.03 [0.99,1.07] p=0.148 | R=1.02 [0.95,1.10] p=0.570 | R=1.00 [0.93,1.07] p=0.973 | R=1.02 [0.95,1.09] p=0.654 | R=1.05 [0.94,1.17] p=0.365 | R=0.76 [0.63,0.91] p=0.004 | R=0.95 [0.88,1.03] p=0.230 | R=1.25 [0.88,1.77] p=0.200 | R=0.97 [0.89,1.07] p=0.548 |
| 2Y | R=1.01 [1.01,1.01] p<0.001 | R=1.01 [0.93,1.09] p=0.825 | R=1.01 [0.97,1.06] p=0.643 | R=1.06 [0.98,1.14] p=0.160 | R=1.03 [0.94,1.12] p=0.556 | R=1.03 [0.95,1.10] p=0.482 | R=1.08 [0.95,1.24] p=0.242 | R=0.84 [0.72,0.97] p=0.022 | R=1.07 [0.97,1.19] p=0.182 | R=1.32 [1.07,1.62] p=0.010 | R=1.04 [0.93,1.16] p=0.517 |
| 3Y | R=1.01 [1.01,1.02] p<0.001 | R=1.00 [0.93,1.07] p=0.959 | R=0.99 [0.95,1.04] p=0.703 | R=1.03 [0.96,1.11] p=0.408 | R=1.01 [0.93,1.10] p=0.781 | R=1.05 [1.00,1.10] p=0.056 | R=1.08 [0.95,1.22] p=0.253 | R=0.85 [0.74,0.98] p=0.024 | R=1.05 [0.95,1.16] p=0.310 | R=1.79 [1.59,2.02] p<0.001 | R=1.07 [0.97,1.19] p=0.184 |
| 4Y | R=0.99 [0.93,1.06] p=0.823 | R=0.98 [0.89,1.08] p=0.701 | R=0.98 [0.93,1.03] p=0.442 | R=0.98 [0.89,1.07] p=0.591 | R=1.04 [0.95,1.13] p=0.382 | R=1.04 [0.95,1.14] p=0.398 | R=1.05 [0.91,1.22] p=0.459 | R=0.78 [0.67,0.90] p<0.001 | R=1.12 [1.00,1.25] p=0.044 | R=1.45 [1.22,1.73] p<0.001 | R=1.13 [1.00,1.27] p=0.048 |
| 5Y | R=1.00 [0.97,1.04] p=0.984 | R=0.99 [0.89,1.09] p=0.783 | R=1.01 [0.96,1.06] p=0.706 | R=1.02 [0.93,1.13] p=0.654 | R=1.06 [0.97,1.16] p=0.183 | R=1.01 [0.92,1.12] p=0.774 | R=1.09 [0.93,1.29] p=0.275 | R=0.82 [0.69,0.99] p=0.036 | R=1.11 [0.99,1.25] p=0.079 | R=1.97 [1.78,2.19] p<0.001 | R=1.03 [0.91,1.18] p=0.629 |
| 6Y | R=1.00 [1.00,1.00] p=0.014 | R=0.95 [0.87,1.05] p=0.307 | R=1.02 [0.96,1.08] p=0.569 | R=1.02 [0.90,1.15] p=0.778 | R=1.04 [0.92,1.18] p=0.537 | R=1.05 [1.01,1.10] p=0.015 | R=1.13 [0.91,1.39] p=0.262 | R=0.79 [0.66,0.94] p=0.009 | R=1.12 [0.96,1.29] p=0.137 | R=2.09 [1.88,2.32] p<0.001 | R=0.93 [0.79,1.09] p=0.360 |
| 7Y | R=1.05 [0.99,1.11] p=0.092 | R=0.91 [0.81,1.03] p=0.132 | R=0.99 [0.92,1.05] p=0.666 | R=1.02 [0.88,1.19] p=0.742 | R=1.05 [0.93,1.19] p=0.432 | R=1.07 [0.97,1.18] p=0.154 | R=1.20 [0.97,1.49] p=0.101 | R=0.63 [0.51,0.79] p<0.001 | R=1.06 [0.93,1.21] p=0.389 | R=1.40 [0.93,2.10] p=0.106 | R=0.99 [0.85,1.14] p=0.865 |
